# Towards high-field applications: high-performance, low-cost iron-based superconductors

**DOI:** 10.1093/nsr/nwae122

**Published:** 2024-03-30

**Authors:** Chiheng Dong, Qingjin Xu, Yanwei Ma

**Affiliations:** Key Laboratory of Applied Superconductivity, Institute of Electrical Engineering, Chinese Academy of Sciences, Beijing 100190, China; Institute of Electrical Engineering and Advanced Electromagnetic Drive Technology, Jinan 250013, China; University of Chinese Academy of Sciences, Beijing 100049, China; Institute of High Energy Physics, Chinese Academy of Sciences, Beijing 100049, China; University of Chinese Academy of Sciences, Beijing 100049, China; Key Laboratory of Applied Superconductivity, Institute of Electrical Engineering, Chinese Academy of Sciences, Beijing 100190, China; University of Chinese Academy of Sciences, Beijing 100049, China

**Keywords:** iron-based superconductors, superconducting wires, critical current density, flux pinning, superconducting joints, high-field magnets

## Abstract

High magnetic fields play a crucial role in advancing basic sciences, fusion energy, and magnetic resonance imaging systems. However, the widespread use of high-field magnets requires affordable high-temperature superconducting wires that can carry large supercurrents. Iron-based superconductors offer an economically attractive solution to push forward important yet costly scientific programs, such as nuclear fusion reactors and next-generation particle accelerators. In this review, we start with the fabrication of iron-based superconducting wires and tapes and continue to discuss several key factors governing the current transport properties. State-of-the-art wires and tapes are introduced with emphasis on grain boundary characteristics, flux pinning, and anisotropy. The architecture of flexible conductors enables low cost, high mechanical strength, and high thermal stability. Recent progress in practical applications, including superconducting joints and insert coils, is also reviewed. Finally, we propose several key questions faced by iron-based superconductors in future practical applications.

## Teaser

Iron-based superconductors have come out from laboratory settings to practical applications as compelling candidates for high-field magnets. This review highlights their latest advancements, essential properties, and critical challenges toward high-performance, low-cost conductors.

## INTRODUCTION

**Figure 1.**
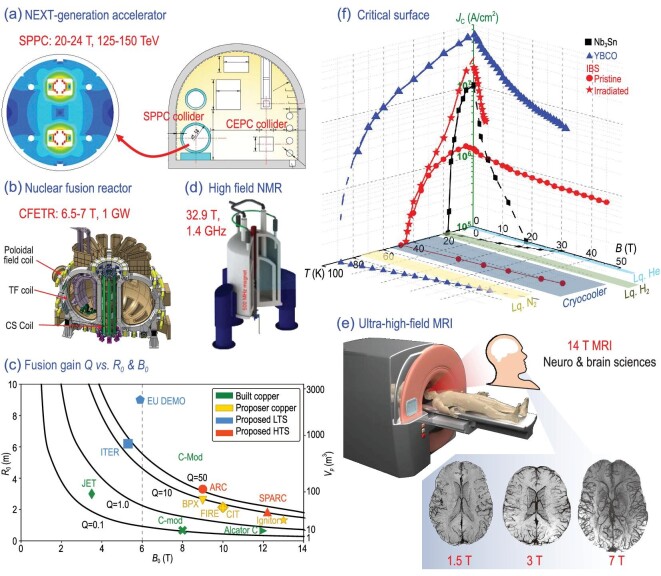
High-field applications of practical superconductors. (a) The next-generation accelerator in China, SPPC. The right shows the tunnel of the accelerator, and the left is the field distribution of the dipole magnets. (b) Nuclear fusion reactor CFETR in China [8]. Copyright 2022, IOP Publishing. (c) Fusion gain Q (black lines) plot against radius *R*_0_ and toroidal field on axis *B*_0_. The plasma volume is shown on the right axis [7]. Copyright 2020, Cambridge University Press. (d) High-field NMR system [9]. Copyright 2021, Springer Nature. (e) Ultra-high-field MRI and brain images obtained at different fields. Higher-resolution images of finer vessels can be obtained at ultra-high fields [10]. Copyright 2018, Elsevier. (f) The critical surface of practical superconductors: Nb_3_Sn, YBCO tapes, and iron-based superconductors (IBS). The basal plane marks the level for practical applications, *J*_c_ = 10^5^ A/cm^2^.

High magnetic fields are powerful tools for precisely manipulating the motion, energy state, and ordering of atoms and charged particles. Due to fundamental electromagnetic interactions, ultra-high magnetic fields are widely applied in basic scientific research, enabling cutting-edge medical technologies, and exploring novel energy resources. For example, the discovery of the Higgs boson [1] at the Large Hadron Collider (LHC, 9 T magnets made from NbTi wires) inspired scientists to further increase the center-of-mass collision energy, which is proportional to the field (*B*) and the radius (*ρ*) of an accelerator *E*[GeV]∼0.3 × *B*[T] × *ρ*[m], from ∼10 TeV to ∼100 TeV. The European Organization for Nuclear Research (CERN) is going to upgrade the accelerator to the high-luminosity LHC operating at 11–13 T [2] and plans to build a 100-km long Future Circular Collider (FCC) working at 16 T. Because the magnetic field is close to the limit of Nb_3_Sn wires used in the dipole and quadrupole magnets of FCC, high-temperature superconducting wires are required to further enhance the collision energy. The scientists from the CEPC-SPPC study group proposed the Super Proton-Proton Collider (SPPC) project aiming at the 125–150 TeV collision energy, which critically relies on the 20–24 T magnets made from high-temperature superconductors [3], as shown in Fig. 1(a). On the other hand, Tokamak magnet systems, e.g. the ITER, are considered to be promising next-generation energy technologies. The combination of the toroidal field (TF) coils and the central solenoid (CS) coils confines the high-temperature plasma in a torus shape (Fig. 1(b)). Because the fusion power is proportional to ∼*R*_0_^3^*B*_0_^4^ [4] (*R*_0_ is the radius of the plasma), compact fusion reactors demand larger magnetic fields, as shown in Fig. 1(c). For example, the China Fusion Engineering Test Reactor (CFETR) will produce over 1 GW of fusion power based on the 6.5–7 T magnets. Bi2212 cables are considered to be employed in the CS coils [5]. The prototype of commercial fusion reactors, SPARC, with the toroidal field on axis of 12.2 T is designed as a compact superconducting tokamak made from REBCO tapes [6,7]. Moreover, the spectral resolution of nuclear magnetic resonance (NMR) systems, as well as the signal-to-noise ratio (SNR) of magnetic resonance imaging (MRI) systems are proportional to the magnetic field, as shown in Fig. 1(e). A 50 μm image resolution is expected on the 14 T MRI, which will considerably promote the study of brain sciences. Therefore, high-temperature superconductors and their applications are of great significance and have revolutionary implications for advancing sciences and technologies.

The application potential of superconductors in high-power devices is not only affected by the intrinsic superconducting properties but also by the difficulty and cost of fabricating long wires. The former is usually described by the critical surface demarcated by the superconducting transition temperature T_c_, the upper critical field *B*_c2_, and the critical current density *J*_c_. As shown in Fig. 1(f), the *J*_c_ of low-temperature superconductors (LTS), Nb_3_Sn, quickly decreases below the level for practical applications ∼10^5^ A/cm^2^ near 20 T due to the low *B*_c2_. On the contrary, YBCO tapes have a larger critical surface area than LTS and the *J*_c_ remains as tens of MA/cm^2^ at 30 T [11]. However, YBCO tapes have not been widely applied in practical applications because the cost per kA-m is currently prohibitively high. Iron-based superconductors (IBS) discovered in 2008 provide a new opportunity in practical applications [12] due to their inherent superconducting properties, e.g. high *B*_c2_∼60 T at 14 K [13], high T_c_ up to 55 K [14], and low anisotropy parameter γ <2 in the 122 system. The red lines in Fig. 1(f) show the critical surface of the 1111-type IBS. According to the *B*_c2_-T curve, the IBS are expected to be used in liquid He, liquid hydrogen, and at temperatures achieved by cryocoolers. Moreover, the *J*_c_ of the 1111 superconductors shows a robust field dependence due to the large *B*_c2_ [15]. For now, the pinning efficiency described by *η* = *J*_c_/*J*_d_ of the pristine 1111-system (*J*_d_∼100 MA/cm^2^) is <1% because of the lack of strong pinning centers. However, the *η* is enhanced to ∼20% (*J*_c_(5 K)∼20 MA/cm^2^) after introducing column defects by irradiation [16,17], as shown by the red stars in Fig. 1(f). (See online supplementary material for a color version of this figure.) Consequently, from the perspective of current carrying ability, IBS are formidable competitors in high-field applications after the flux pinning structure is well constructed.

**Figure 2.**
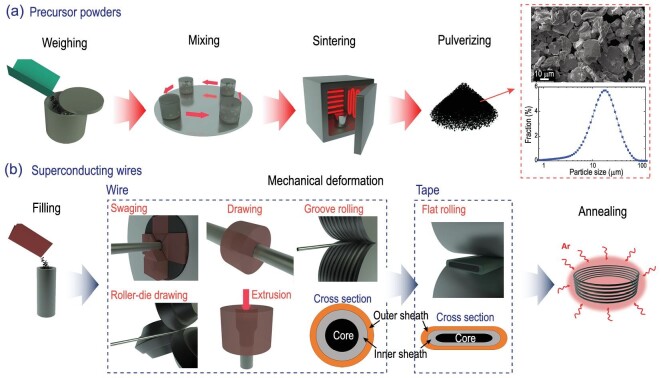
Fabrication procedures of the (a) iron-based superconducting precursors, (b) iron-based superconducting wires and tapes. The upper figure in the red box is the scanning electron microscopy (SEM) image of the precursor powders. The figure below depicts the distribution of the particle size. The peak is located at *d*∼18 μm [18]. The transverse cross-sections of the wires and tapes are depicted at the bottom of (b).

LTS belong to intermetallic compounds with good ductility. They can be directly fabricated into long wires by mechanical deformation processes. Instead, IBS are semimetals with elements assembled by ionic or covalent bonds. They behave more like ceramics with bad ductility. One efficient way to make them into long wires is to follow the routes of Bi2223 and Bi2212 conductors, namely the powder-in-tube (PIT) method [19–21]. The precursor powders fabricated by the *ex-situ* method are filled in a metal tube which is then subjected to a series of mechanical deformations, as shown in Fig. 2(b). For example, swaging, groove rolling [22], roller-die drawing [23], extrusion [24], and drawing are usually applied to make round wires. Flat rolling is used to transform the wires into tapes, as shown by the schematic transverse cross-sections depicted in Fig. 2(b). The final high-temperature sintering is indispensable to recrystallizing grains, repairing cracks, and better grain connectivity. It must be emphasized that the toxic As is handled in a glove box during the precursor fabrication, and it becomes chemically stable after heat treatment. The IBS wires and tapes remain harmless during the winding and impregnation of magnets due to the protection afforded by the metal sheaths. For Bi-based conductors, silver or silver-alloy sheaths are the only choices before annealing in an atmosphere with controlled oxygen pressure. This is necessary to facilitate the diffusion of oxygen atoms for controlling superconductivity. On the contrary, iron-based superconducting wires and tapes can be directly annealed in a vacuum or inert atmosphere, providing more options for the sheath materials.

## PRECURSOR POWDERS: CORNERSTONES OF LARGE-SCALE PRODUCTION OF HIGH-PERFORMANCE SUPERCONDUCTING WIRES AND TAPES

As the starting materials of the PIT process, iron-based superconducting precursors are the foundation of producing long wires. The quantity and quality of the powders directly determine the length and performance of the superconducting wires. In addition to the intrinsic superconducting properties, the precursors must have three essential attributes:

1. easy fabrication process,
2. high purity and homogeneity,
3. chemical inertness to environments and metal sheaths.

So far, many kinds of IBS have been discovered. However, only a few of them are suitable for the PIT process. SmFeAsO_1-x_F_x_ superconductors have a high superconducting transition temperature at T_c_∼55 K [25]. But impurities, such as Sm_2_O_3_ and FeAs, are difficult to remove in the precursor bulks, leading to a small *J*_c_∼10^4^ A/cm^2^ of wires and tapes at 4.2 K and 10 T [26]. For the FeSe11 and CaK1144 systems, the Se, Ca and As elements react with most kinds of metal sheaths, making it a challenging job to enhance *J*_c_ [27–29]. Generally speaking, the metal reacting with arsenic cannot be used as the inner sheath. The reaction layers ∼10–30 μm thick were found at the interface between superconducting filaments and the Nb, Ta, Fe, Cu [30] sheaths. Similar to the Bi-based PIT wires and tapes, silver or silver alloy is the best inner sheath material because of its inertness to Ae_1-x_K_x_Fe_2_As_2_ (AeK122, Ae = Ba, Sr) compounds and good ductility.

**Figure 3.**
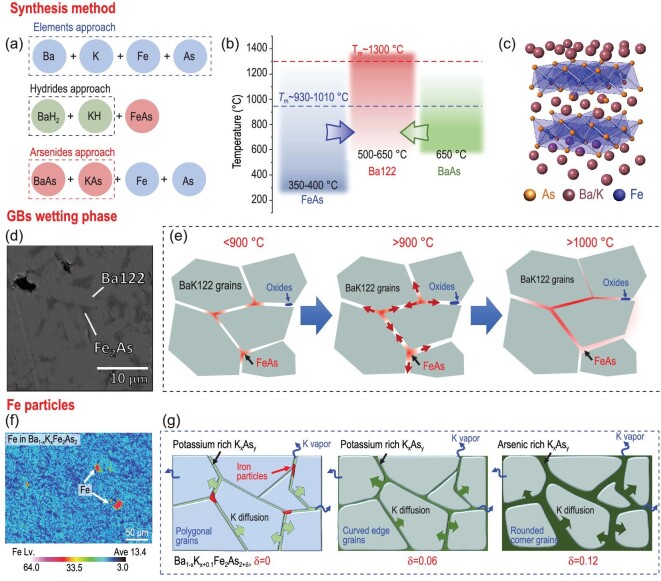
Formation mechanism of Ba_1-x_K_x_Fe_2_As_2_ and impurities. (a) Three synthesis strategies from different starting materials. (b) Phase diagram explaining the formation of the Ba122 phase. The red and blue dashed lines denote the melting temperature, T_m_, of the Ba122 phase and the FeAs, respectively. (c) Ba_1-x_K_x_Fe_2_As_2_ crystal lattice. (d) Fe_2_As impurity phase in the Co-doped Ba122 sintered at 1000°C [42]. Copyright 2021, IOP Publishing. (f) Elemental mapping showing the excess Fe particles in Ba_1-x_K_x_Fe_2_As_2_ precursors [43]. Copyright 2023, Elsevier. Schematic diagram showing (e) FeAs gradually wets the GBs with the increasing temperature, (g) origin and elimination of the excess Fe particles via slightly excessive addition of As. The evaporated intragrain K at high temperatures is counterbalanced by the diffusion from the ‘ocean’ of inter-grain K_x_As_y_.

After one decade of research, it was found that the K-doped FeAs122 precursors are of high purity and chemical stability [31]. Consequently, AeK122 compounds prevail over other IBS and stand out as a model system to be used in PIT wires and tapes. Therefore, we will focus on the AeK122 system in this review. Up to now, different synthesis routes of AeK122 polycrystalline bulks have been proposed, as shown in Fig. 3(a). The commonly used raw materials are Ba/Sr and K bulks, As chunks, and Fe powders. The elemental materials are distinct in basic physical and chemical properties, making it difficult to mix them homogeneously. Moreover, Ba and K are easily oxidized, even in a glove box with an Ar atmosphere. It inevitably brings in noxious oxides into the grain boundaries (GBs) and the inhomogeneous superconducting phase. One effective way is to use the intermediates as the starting materials. Dong *et al.* used the pre-synthesized BaAs and KAs intermediates as the starting materials, not only to avoid oxidation but also to enhance the mixing efficiency of Ba and K in the powder form [32]. The nominal composition Ba_0.6_K_0.5_Fe_2_As_2_ with the excess K was used to compensate for the evaporated K. Zaikina *et al.* utilized hydrides KH and BaH_2_ as the intermediates and successfully synthesized the Ba_1-x_K_x_Fe_2_As_2_ precursors [33]. The salt-like hydrides facilitate the thorough mixing of the starting powders within a short time. More importantly, the evaporated hydrogen provides a reducing atmosphere to prevent oxidation of the highly reactive elements. However, more precautions must be taken on handling highly reactive metal hydride intermediates.

The starting materials are generally mixed by a high-energy planetary ball-milling machine [34] or a high-impact shaker mill [29,35,36]. The milling balls impact the powders at a high speed, flattening and smashing the hard materials and cold-welding them to form the intermediates. It even initiates the partial formation of the superconducting phases, enabling final sintering at low temperatures. The thoroughly mixed powders are then sintered at high temperatures in a vacuum or inert atmosphere. Traditional sintering methods at ambient pressure (AP) are prevalent in most research groups. The mixing powders are pressed into pellets and sealed in a stainless steel (SS) tube which is then heated up to 800–900°C under an Ar atmosphere for <24 hours. The hot-isostatic-pressing (HIP) method was also applied to the raw materials [37,38]. It is suggested that sintering at temperatures well below the melting temperature of FeAs is helpful in increasing the inter-grain current transport. Spark plasma synthesis (SPS) applies high pulsed currents passing through a compact sample pressed under a high uniaxial pressure of 50–100 MPa. The formation of the superconducting phase can be finished in 5–20 minutes after fast heating up to the desired temperatures [39,40]. Another rapid synthesis method, fast combustion synthesis, was used to fabricate the FeSe and FeTe_1-x_Se_x_ compounds [41]. Under an Ar atmosphere, a current of 10 A was applied in the tungsten coil above the pre-mixed powders for 2 seconds to ignite the reaction. The following reaction continues in a self-sustained way. However, the application of this method for the massive production of AeK122 compounds still needs further investigation.

The phase formation mechanism of the AeK122 precursors is vitally important to understand the sequence of the chemical reaction and shed light on methods to avoid impurity phases. Based on the differential scanning calorimetry (DSC) measurements, Tokuta *et al.* concluded that the formation of FeAs occurs at 348°C before the appearance of the Ba122 phase at 640°C [42]. With the help of mechanical alloying during the ball milling process at a high speed of 800–1000 rpm, they even obtained a pure Ba122 phase at a relatively low temperature of 600°C. Zaikina utilized the *in-situ* high-temperature synchrotron powder X-ray diffraction to study the phase formation mechanism of BaFe_2_As_2_ using BaH_2_, Fe and As as the starting materials [40]. The decomposition of BaH_2_ begins at 311°C, while the FeAs appears at 385°C. The FeAs content increases with temperature. At 512°C, the Ba element or the BaAs react with FeAs to form BaFe_2_As_2_, as shown by Fig. 3(b). It indicates that the AeFe_2_As_2_ phase can be obtained at temperatures as low as 500–600°C. Therefore, there is a large reaction temperature window to obtain the AeK122 superconducting phase.

Even though the as-synthesized AeK122 precursor bulks exhibit a single-phased X-ray diffraction pattern [44], there are still secondary phases beyond the detection limit of traditional spectroscopic devices. Scientists have utilized instruments with a higher spatial resolution to focus on the detailed characteristics near GBs. Kim *et al.* applied atom-probe tomography (APT) to study the GBs composition of the K- and Co-doped BaFe_2_As_2_ compounds [45]. They found that all the studied GBs have significant compositional variations at the scales of ∼10 nm, which is ∼2–3 times the coherence length, ξ. They suggested that AsO and Ba-rich oxide brought by the starting materials are the preponderant impurities at GBs. Kametani *et al.* investigated the nanostructure and chemical variations at GBs of the K-doped BaFe_2_As_2_ bulks by scanning transmission electron microscopy (STEM) [46]. It was found that oxides of Ba and K are the major contamination existing in the GBs. To eliminate these oxides, Pak *et al.* started from the commercially available elemental raw materials with the highest purity: Ba/K (≥99.9%), Fe (≥99.99%) and As (≥99.99999%) to fabricate Ba_1-x_K_x_Fe_2_As_2_ bulks [47]. The chemical handling procedures were carried out in a high-performance glove box with O_2_ ≤0.005 ppm and H_2_O ≤0.06 ppm. Combined with HIP at 600°C, they obtained high-quality samples with fewer FeAs and BaO impurities at GBs.

The studies mentioned above all claimed the absent FeAs because of the lower sintering temperature (HIP, 600°C) than the melting point of FeAs (*T*_m_∼930–1010°C). But for the IBS bulks sintered at higher temperatures, the FeAs wetting phase appears between grains [42], as shown by Fig. 3(d). More importantly, the FeAs wetting phase existing in the precursor bulks will be brought into wires and tapes. One possible origin of the GBs wetting phase is proposed in Fig. 3(e). Because FeAs or Fe_2_As act as the sources for the formation of the Ba122 phase, the local non-equilibrium chemical ratio may lead to redundant FeAs between grains. Sintering at temperatures higher than the *T*_m_ of FeAs will cause them to melt and gradually wet the GBs. However, further evidence is still needed to prove this hypothesis.

Another impurity commonly seen in IBS wires and tapes is the 10–30 μm sized excess Fe particles [22,48–50], as shown by the element mapping in Fig. 3(f). They not only behave as current blockers, especially in filaments with small sizes, but also provide strong magnetism and cause thermal turbulence. Tu *et al.* systematically studied the origin of the excess Fe particles by modifying the nominal composition Ba_1-x_K_x+0.1-β_Fe_2_As_2+δ_ [43]. It is found that the excess Fe appears because the stoichiometric As is exhausted by the preferential reaction with the excess K in the commonly used nominal composition Ba_0.6_K_0.5_Fe_2_As_2_, leaving the unreacted Fe between the grains. Reducing the excess K content is efficient in reducing the excess Fe but also suppresses superconductivity. On the contrary, slightly over-adding As (δ∼0.04) is found to be a twin-track approach to diminish the excess Fe and preserve superconductivity, as shown in Fig. 3(g). However, higher δ >0.06 prohibits grain growth (rounded corner grains) and introduces new inter-grain impurities largely deteriorating the grain coupling.

## INTER-GRAIN COUPLING: THE NECESSARY PATH TO HIGH *J*_C_ CONDUCTORS

**Figure 4.**
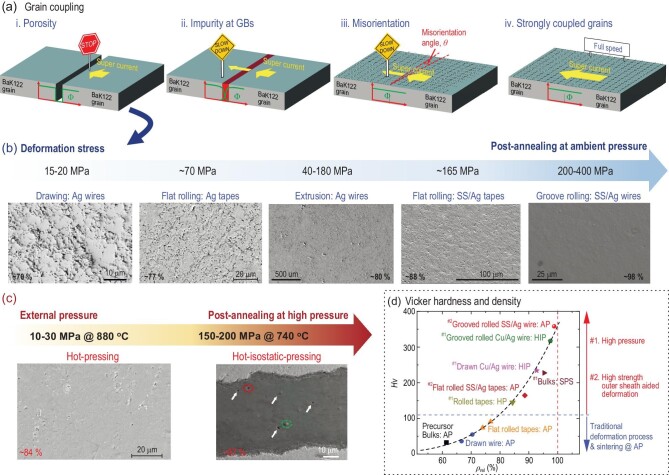
(a) Grain coupling issues in BaK122 wires and tapes. Green curves show the variation of the superconducting order parameter, Φ, across the GBs. The yellow arrows are the supercurrents. The *ab*-plane of the crystal lattice is shown by the black grids in iii and iv. Two different ways to enhance core density: (b) cold deformation and annealing at AP, and (c) high-pressure sintering. The deformation stress and the external pressure are marked on the blue and red arrows, respectively. The microstructures along with the relative core density are shown below [22,49,53,54]. Copyright 2023, Elsevier; Copyright 2021, Springer Nature; Copyright 2014, Springer Nature; Copyright 2015, IOP Publishing. (d) Relation between the relative core density *ρ*_rel_ = *ρ*_mea_/*ρ*_theory_ and the Vickers Hardness Hv of superconducting cores. *ρ*_mea_ and *ρ*_theory_ are the measured and theoretical core density, respectively. The red dashed line denotes 100% limit (*ρ*_theory_ = 5.86 g/cm^3^ for Ba_0.6_K_0.4_Fe_2_As_2_), and the blue line separates the special densification techniques from traditional fabrication methods.

The fundamental basis for the current transport in polycrystalline PIT wires and tapes is the grain coupling, as illustrated in Fig. 4(a).

1. ***Porosity.*** In porous polycrystals, micro gaps between grains render a high energy barrier to charge carriers and suppress the superconducting order parameter Φ to zero, as shown in Fig. 4(a)i. The macroscopic cracks and voids in superconducting cores decrease the effective cross-section areas of supercurrent and substantially suppress *J*_c_. As a result, improving the superconducting core density has become one of the major tasks in recent years.
2. ***GBs impurities.*** As mentioned above, the GBs of IBS are commonly found to be contaminated by the FeAs wetting phase, as shown in Fig. 4(a)ii. The transparency of GBs for supercurrent depends on the thickness and electronic properties of the GBs secondary phase. If the superconducting order parameter, Φ, is partially preserved in the FeAs phase due to the proximity effect [44], the overlap of Φ in GBs would permit part of the supercurrent to cross GBs.
3. ***Weak-link effect.*** Even if the grains are closely connected without GBs impurities, the inter-grain critical current density is largely depressed when the misorientation angle is larger than a critical value, θ_c_∼9^o^ [51], as shown in Fig. 4(a)iii. This value is better than the θ_c_ = 5^o^ of cuprates. However, improving the grain texture is still a formidable challenge for IBS wires and tapes.
4. ***Strongly coupled grains.*** Closely connected grains with clean GBs and small misorientation angle θ <9^o^ are the best case in IBS wires and tapes, as depicted in Fig. 4(a)iv. The supercurrent will be maintained or even enhanced in a small angle GBs network [52].

Fig. 4(b) shows the calculated deformation stress via finite element analysis (FEA) [24]. Drawing introduces the radial compressive stress in addition to the axial tensile stress. However, the lateral compressive stress induced by area reduction only achieves 5–10 MPa for the Ag-sheathed wires. On the contrary, the axial tensile stress is over 20 MPa, which may stimulate propagating cracks and reduce the relative core density below 70%. The corresponding SEM image shows large amounts of voids in the transverse cross-section of the as-drawn Ag wire sintered at AP. Consequently, the as-drawn wires usually present a low *J*_c_. Through the HIP method with pressure up to 150–200 MPa, the wire uniformly contracts in the radial direction. The voids and cracks in the as-drawn wire are conspicuously diminished, as shown in Fig. 4(c). The relative core density is enhanced to nearly 100% [37]. Extrusion applies a typical triaxial compressive stress to the superconducting core [24]. The calculated stress is over 30 MPa, much larger than the radial stress of drawing. Moreover, if a large reduction rate is applied, the compressive stress escalates to 180 MPa. The relative core density is largely improved, and the voids and cracks are considerably reduced. Dong *et al.* applied groove rolling to a SS/AgSn/Ag Ba_1-x_K_x_Fe_2_As_2_ round wire [22]. Due to the high yield stress of the work-hardened SS, the wire requires a larger force for deformation. The groove roller transmits a nearly isotropic transverse compression stress to the inner cores. The transient stress is as large as 470 MPa, and the final relative core density approaches 100%. The cross-sectional SEM image barely shows any voids or cracks. This easy fabrication process no longer depends on expensive HIP devices but achieves the same compaction effect. It can be scaled up to long wire productions.

The IBS wires are further flat-rolled into tapes to introduce partial *c*-axis texture. Flat rolling applies a uniaxial compressive press of ∼70 MPa to the tape under the flat roller, leading to an enhanced core density. However, there are still many voids and cracks in the SEM images. Hot-pressing (HP) applies a small pressure of 15 MPa to the tape at 880°C but considerably diminishes the porous structure, as shown in Fig. 4(c) [55]. This is caused by the thermal extension of the superconducting core along the transverse direction during the thermal-mechanical deformation process. However, HP is not suitable for long tape productions. The HIP method was also applied to the sintering of the Cu/Ag composite tape and achieved a nearly 100% dense core [49]. This manufacturing process can be applied in kilometer-long tapes. The SS/Ag composite structure was also used in tape productions. To prevent large deformation at the center of the core, the Ag tape was first rolled into the desired dimensions and then inserted into a pre-rolled SS tube before the flat rolling process. According to the FEA results, the stress in the core is as high as 165 MPa, resulting in a relative density of 88%. Compared with the flat-rolled Ag tape, the voids in the SS/Ag tapes are considerably reduced.

To summarize, the traditional deformation process of the Ag tape and sintering at AP cannot achieve a dense superconducting core. There are two ways to solve this problem: high-pressure sintering and high-strength outer sheath aided deformation. The dependence of the Vickers hardness Hv on the relative core density is shown in Fig. 4(d). All the data fall into a single curve described by Hv = 375.55*ρ_rel_^5.332^, where *ρ*_rel_ is the relative core density [24]. For the precursor bulks sintered at AP, it only achieves 61% of the theoretical density *ρ*_theory_. Subsequent traditional deformation processes of the Ag wire, including swaging, drawing, and flat rolling, slightly increase the *ρ*_rel_ to 77%. High-pressure sintering increases the *ρ*_rel_ to 84% for the hot-pressed Ag tape and 95% for the HIP-processed Cu/Ag round wire. The *ρ*_rel_ of the flat-rolled SS/Ag tape sintered at AP is 88%, better than that of the HP tapes. The highest Hv ever achieved in Ba_0.6_K_0.4_Fe_2_As_2_ superconductors is ∼360 in the groove-rolled SS/Ag wire sintered at AP [22]. The corresponding density is close to the theoretical value.

**Figure 5.**
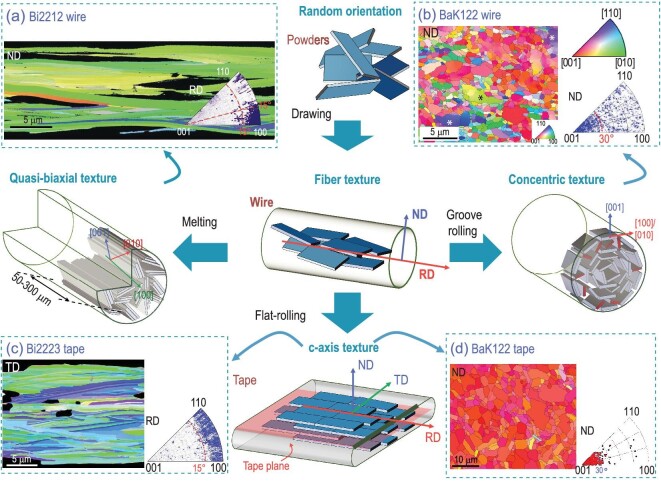
Grain texture of practical superconducting wires and tapes: (a) Bi2212 wires [56], (b) BaK122 wires [22], (c) Bi2223 tapes [56], and (d) BaK122 tapes (HP) [57]. Copyright 2015, Springer Nature; Copyright 2023, Elsevier; Copyright 2021, IOP Publishing. The cartoons show the evolution of grain alignments during the PIT process. In the blue boxes, the inverse-pole-figure (IPF) maps are shown on the left, and by the side are the corresponding IPF. The IPF map is colored according to the crystallographic triangle at the top right of (b).

Before the post-annealing of PIT wires and tapes, the sizes and alignments of grains are mainly determined by the mechanical deformation. It is found that the BaK122 grain size decreases from 16 μm of the starting precursor powders to 10 μm after the drawing with a large reduction rate [22]. Moreover, the isotropic compressing stress and axial tensile stress of drawing make the plate-like grains align along the wire axis and form the fiber texture, as shown in the middle of Fig. 5. The larger deformation force applied by the following groove rolling violently crushes the grains to a size of 3–5 μm in the round wires. Instead, the uniaxial compression in flat-rolling and HP aligns the basal plane of grains parallel to the tape surface. On the other hand, the grain size and alignments are also controlled by the growing conditions at high temperatures. For the Bi2212 round wires, the grains crystallize from the melt under sparse nucleation conditions. The confined space of the Ag cavity ∼15 μm in diameter compels the *a*-axis of the grains to expand at a much faster rate along the wire axis than the *c*-axis and captures off-axis grains produced by random nucleation. While the growth of the *b*-axis is greatly limited by the transverse direction of the filament. Consequently, one can find long plate-like grains extending along the filament with 0.3–1 μm in thickness, 5 μm in width and 50–300 μm in length, as shown in Fig. 5(a). The in-plane *a*/*b* aspect ratio is larger than five [56]. For the Bi2223 tape, after the multiple rolling and reaction induced alignments of the Bi2212 grains, there occurs the formation of Bi2223 grains via solid-state diffusion with a very small amount of liquid phase involved. The filament of the Bi2223 tape is ∼230 μm wide and 10 μm in thickness, allowing free development of the *a*- and *b*-axis in any direction within the *ab*-plane. The thickness and diameter of the round shaped 2223 grains are 0.3–1 μm and 5–20 μm, respectively. The grains of Bi2212 wires and Bi2223 tapes prefer to stack on top of each other to constitute a colony structure sharing the same *c*-axis. Unlike the Bi-based cases, the post-annealing temperatures (T_ann_∼600–900°C) of BaK122 wires and tapes are usually far below the melting temperature. Therefore, it only concerns the solid-state reaction rather than recrystallization from the melt. The average size of the grains in BaK122 flat-rolled tapes sintered at 900°C AP is 0.5–1.5 μm in thickness and 2 μm in diameter [57]. There are also a few grains larger than 5 μm. However, parts of the small grains in the flat-rolled tape recrystallize into larger ones (5–6 μm) after HP. Comparatively, the SS-sheathed wires and tapes go through a large compressive stress which pulverizes the grains into submicron particles (300–700 nm) [22,44], as shown in Fig. 5(b). The following sintering at a relatively low temperature of ∼750°C is not high enough to recrystallize them into large grains.

The texture of superconducting filaments is usually investigated by XRD or the electron backscatter diffraction (EBSD) technique. Kametani *et al.* performed a detailed study on the texture of Bi-based wires and tapes via EBSD [56]. They concluded that the *a*-axis of the long slab like Bi2212 grains aligns along the wire axis, as shown by the normal directional (ND) inverse-pole-figure (IPF) map in Fig. 5(a). The corresponding RD IPF on the right shows a narrow spread of the in- and out-of-plane misorientations, suggesting a quasi-biaxial texture [58]. The largest misorientation angle between [100] and the wire axis is 15^o^. It is suggested that the [010] and [001] directions rotate around the wire axis, as shown by the schematic diagram below. The brick wall model predicts a supercurrent along the [001] twist GBs if the in-plane current is blocked. It applies well to Bi2212 wires due to the large basal plane of the stacked grains providing a strongly linked current path with a small misorientation angle [59]. The texture of BaK122 wires is different. The average size of the filament is 230 μm [22]. The grains larger than 1 μm are subjected to the radial compressing force and lie down on the surface perpendicular to that force. While the orientation of the submicron grains is quite random. As shown by the IPF in Fig. 5(b), the grain orientation points cluster within 30^o^, indicating partial *c*-axis texture. But there is no in-plane texture developed. Pyon *et al.* performed XRD measurements on the longitudinal cross-section of a single filament Cu/Ag round wire and concluded the concentric texture [60]. Considering the nearly 100% core density, the low texture is considered to be the main obstacle to the *J*_c_ enhancement of round wires.

The situations of the Bi2223 and BaK122 tapes are similar. The uniaxial compressive stress of flat-rolling causes lateral extension of the transverse cross-section. The grains tend to lie on the tape plane to form a *c*-axis texture. The IPF in Fig. 5(d) manifests that the out-of-plane misorientation angles *θ*_OFP_ are up to 30^o^ in the BaK122 tapes. Comparatively, the *θ*_OFP_ of Bi2223 tapes is limited to 15^o^, indicating better *c*-axis texture than that of BaK122. It may benefit from the multiple rolling and intermediate annealing process. The [100] and [110] directions uniformly distribute in the RD IPF without obvious direction, indicating no in-plane texture is present. The rounded grains of the Bi2223 and BaK122 wires and tapes are much smaller than those of the Bi2212 wires, implying that the brick wall model may be inappropriate. The supercurrent could primarily run within the *ab*-plane as predicted by the railway switch model, which proposes a dominant in-plane supercurrent across the small angle [001] tilt GBs. However, further evidence on the intrinsic current transport mechanism of BaK122 wires and tapes is still lacking.

**Figure 6.**
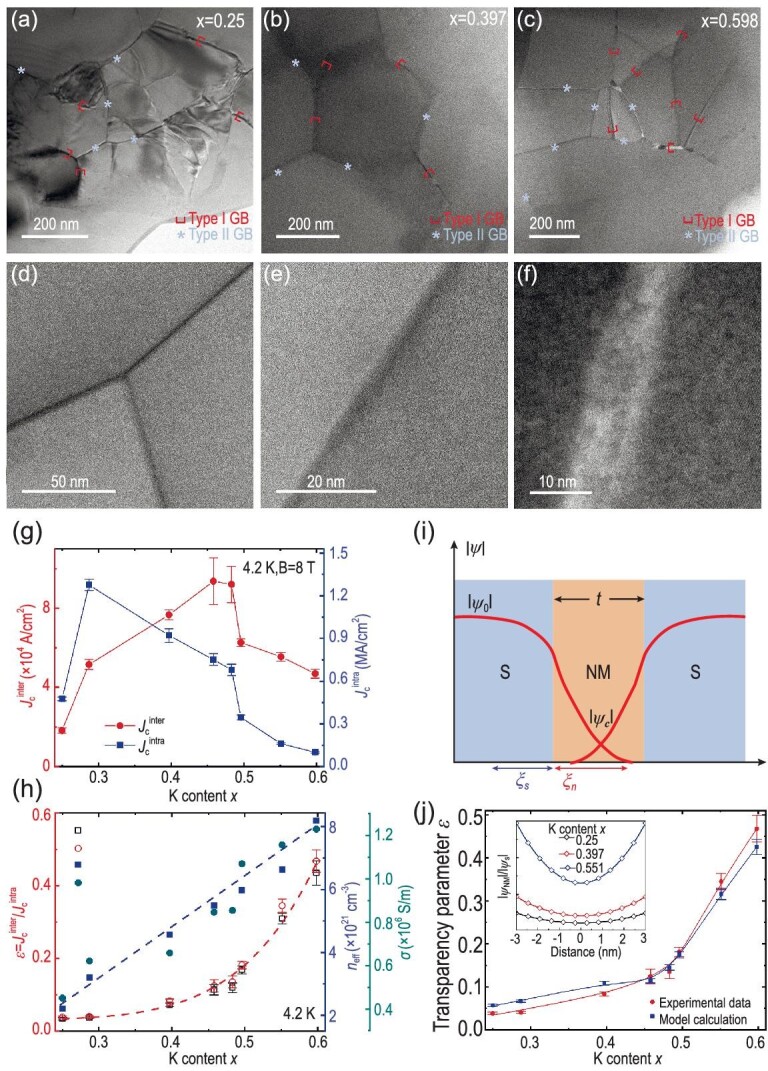
*Dirty GBs* characteristics in Ba_1-x_K_x_Fe_2_As_2_ tapes [44]. Copyright 2022, Elsevier. (a–c) GBs of *x* = 0.25, 0.397 and 0.598. Two types of GBs can be found, despite the K doping content: (d) and (e) Type-II GBs, (f) type-I GBs. Doping dependence of (g) intra- and inter-grain *J*_c_, (h) GBs transparency parameter *ε* = *J*_c_^inter^/*J*_c_^intra^, effective charge carrier density *n*_eff_ and conductivity σ. (i) SNS Josephson junction (S: BaK122 superconductor, N: FeAs normal metal). The red line denotes the superconducting order parameter: $| {{{\psi }_0}} |$ in the grains and $| {{{\psi }_c}} |$ in the GBs. (j) Calculated GBs transparency parameter. The inset shows the calculated normalized superconducting order parameter in GBs.

Impurities at GBs are the archenemy of the *J*_c_ of high-temperature superconductors. Fast cooling from the melt generates amorphous phases at GBs that cannot re-solidify into the Bi2212 grains [58], resulting in a lower *J*_c_ than that of the wires with cleaner GBs but worse textures. The same issue also presents in IBS. Cheng *et al.* doped the SS/Ag sheathed Ba_1-x_K_x_Fe_2_As_2_ composite tapes in a wide range (0.25 ≤ *x* ≤ 0.598) to study the effect on inter-grain transport properties. It is found that most GBs are contaminated by the FeAs phase, regardless of the doping level. As shown in Fig. 6(a–c), two types of GBs randomly distribute in the tapes: type I with thickness *t* larger than 5 nm (∼2ξ), and type II with *t* ≤ 5 nm. Fig. 6(g) exhibits the doping dependence of the intra-grain *J*_c_^intra^ and inter-grain *J*_c_^inter^. Similar to the BaK122 single crystal, the *J*_c_^intra^ peaks at the slightly under-doped level *x*∼0.287 due to the stronger intragrain flux pinning provided by the AFM/structural domain boundaries [61,62]. Unexpectedly, the *J*_c_^inter^ reaches its maximum at the slightly overdoped level of *x*∼0.458. The GBs transparency parameter defined by *ε* =* J*_c_^intra^/*J*_c_^inter^ continuously increases with *x*, along with the improved effective hole density *n_eff_* and the electrical conductivity σ, as shown in Fig. 6(h). The commonly used optimal doping *x* = 0.4 only achieves a *ε*∼8.3%. It promptly increases to 46.8% at *x*∼0.6. It resembles the case of Bi2212 wires [63] and YBCO tapes [64,65]. However, the doping effect of cuprate conductors is based on clean GBs [66]. Instead, the superconductor-FeAs normal metal-superconductor structure in BaK122 tapes can be considered as an SNS Josephson junction. Due to the proximity effect, the superconducting order parameter ${{\psi }_0}$ extends to the FeAs phase with a characteristic length *ξ*_n_, as sketched in Fig. 6(i). The overlap of ${{\psi }_c}$ in the FeAs layer enables the current transport. The calculated normalized order parameter ${{\psi }_c}/{{\psi }_0}$ in the inset of Fig. 6(j) indicates that the ${{\psi }_c}$ increases with K doping. The calculated *ε* fits well to the experimental data. This study suggests that the poisoned GBs can be largely ameliorated by hole doping in the BaK122 grains. However, clean GBs with small misorientation angles are always the best case for current transport.

## CRITICAL CURRENT DENSITY, ANISOTROPY, VORTEX PINNING AND DYNAMICS IN BaK122 WIRES AND TAPES

**Figure 7.**
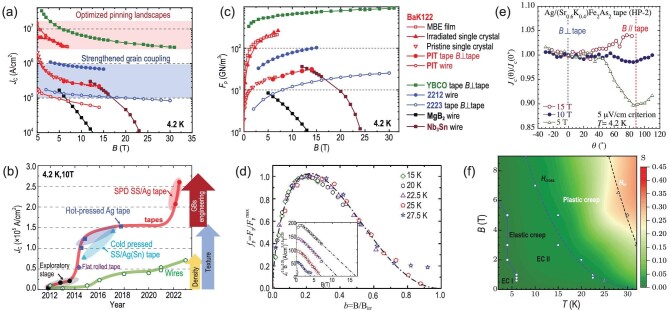
Field dependence of (a) *J*_c_ and (c) flux pinning force density *F*_p_ = *J*_c_ × *B* of practical superconducting wires and tapes at 4.2 K (the data of copper-oxides superconducting wires and tapes are derived from the Non-stabilizer *J*_c_-*B* PLOT by Peter Lee). The legends are shown on the right of (c). The red symbols correspond to BaK122 superconductors [52,67,68]. The *J*_c_ of the BaK122 MBE films and the single crystals were obtained at 4 K and 5 K, respectively, with fields perpendicular to the *ab*-plane. The light blue and light red areas mark the upside potential via strengthening grain coupling and optimizing flux pinning landscapes, respectively. (b) The advancement of *J*_c_ of IBS wires (green line) [37,60,68–74] and tapes (red line) [32,54,55,75–78] with time. (d) Normalized flux pinning force density *f*_p_ = *F*_p_/*F*_p_^max^ as a function of reduced field *b* = *B*/*B*_irr_ [79]. Copyright 2016, AIP Publishing. (e) Angle dependence of normalized critical current density *J*_c_(θ)/*J*_c_(0^o^) at 4.2 K [80]. Copyright 2017, IOP Publishing. (f) Vortex phase diagram marked with magnetization relaxation rate, *S* = dln(-*M*)/dln*t* [81]. Copyright 2019, IOP Publishing.

Figure 7(a) summarizes the *J*_c_(*B*) curves of practical superconducting wires and tapes at 4.2 K. The BaK122 wires and tapes with the highest *J*_c_ are shown by the red circles. At 4.2 K and 10 T, the $J_c^ \bot $ of the BaK122 SS/Ag tapes fabricated by the severe plastic deformation (SPD) method is 2.6 × 10^5^ A/cm^2^. It is larger than Nb_3_Sn and MgB_2_ wires as well as Bi2223 tapes at high fields. However, there is still a large distance to that of 2212 wires, not only because of the T_c_ difference but also due to the better texture of Bi2212 wires. After the grain coupling issue was resolved, the *J*_c_ of the BaK122 wires and tapes could be enhanced to 1 MA/cm^2^ at high fields, as shown by the blue region in Fig. 7(a). Furthermore, the distance to YBCO tapes could be shortened by optimizing flux pinning landscapes. Notably, there have already been two examples of high *J*_c_ near or exceeding 10^7^ A/cm^2^. One is the irradiated BaK122 single crystals with column pinning centers introduced by irradiation [67], and the other is the BaK122 film with a low-angle GBs network acting as strong pinning sources [52]. It is suggestive of a large potential for the *J*_c_ enhancement of BaK122 wires and tapes. Figure 7(b) presents the development of *J*_c_(4.2 K, 10 T) of IBS wires and tapes. The *J*_c_ of the round wires (green line) was largely enhanced above 5 × 10^4^ A/cm^2^ due to the improved core density by HIP and continues to reach 7.1 × 10^4^ A/cm^2^ after enhancing texture [68]. For the IBS tapes (red line), uniaxial pressing or flat-rolling synergistically increases the texture and density of the superconducting core. But the *J*_c_ saturates after 2018 because of the unsolved dirty GBs issue. Recently, clean GBs were obtained in the SPD tapes via GBs engineering, and the *J*_c_(4.2 K, 10 T) is increased to 2.6 × 10^5^ A/cm^2^, as shown by the red areas in Fig. 7 (b). The highest flux pinning force density *F*_p_ of BaK122 tapes is over 30 GN/m^3^ at 14 T, exceeding that of Bi2223 tapes and Nb_3_Sn wires at high fields, as shown in Fig. 7(c). Comparatively, the *F*_p_ is ∼100 GN/m^3^ and 1 TN/m^3^ for 2212 wires and YBCO tapes, respectively. Further enhancement of *F*_p_ can be realized by optimizing flux pinning structures, e.g. irradiation. (See online supplementary material for a color version of Figure 7.)

The flux pinning mechanism of high-performance HP tapes was investigated based on the Dew-Hughes model. The normalized *F*_p_ peaks near *h*_max_∼0.2, as shown in Fig. 7(d), indicating flux pinning by surface defects [79]. Based on the temperature dependence of *J*_c_, Zhuang *et al.* found signs of pinning from the spatial fluctuation of charge carrier mean free path, Δ*l* [82]. Combined with the cumulative TEM evidence of dislocations, it is suggested that the flux pinning may be dominated by dislocations or low-angle GBs. Flux motion causes measurable dissipation and deterioration of *J*_c_. It can be quantitatively evaluated by measuring the time dependence of magnetization, *S* = dln(−*M*)/dln*t*. Figure 7(f) summarizes the vortex phase diagram of HP BaK122 tapes [81]. At low temperatures, the relaxation rate is close to 10^−3^, indicating slow flux motion benefiting from strong flux pinning. With the increasing temperature and field, there occurs a crossover from elastic to plastic flux motion regions at the crossover field *H*_cross_. It is found that the elastic creep region is expanded with the decreased grain size [79]. Inverse dependence of *J*_c_ on grain sizes was also observed in the untextured wires and bulks [83,84].

Generally, the anisotropy of *J*_c_ follows that of H_c2_ determined by the effective mass of electrons in the form of γ = (*m_c_*/*m_ab_*)^1/2^ = (*H_c2_^ab^*/*H_c2_^c^*), where *c* and *ab* denote the field directions along the *c*-axis and the *ab* plane of the crystal lattice. A low anisotropy parameter γ<2 was found at any temperature and field [55,80], much lower than that of cuprate superconducting tapes, as shown in Fig. 7(e). However, *J*_c_^*c*(⊥tape)^*>J*_c_^*ab*(//tape)^ has been found in high-performance IBS tapes [48], which is opposite to the anisotropy of *H_c2_*. Awaji *et al.* ascribed the field-dependent anisotropy to the transition between the unoccupied and the occupied states of pinning centers, namely the dislocation network. The temperature dependence of anisotropy can be explained by the competition between different pinning mechanisms at different temperatures [80].

## CONDUCTOR BY DESIGN: THE KEY TO HIGH-STRENGTH, LOW-COST PRACTICAL CONDUCTORS

**Figure 8.**
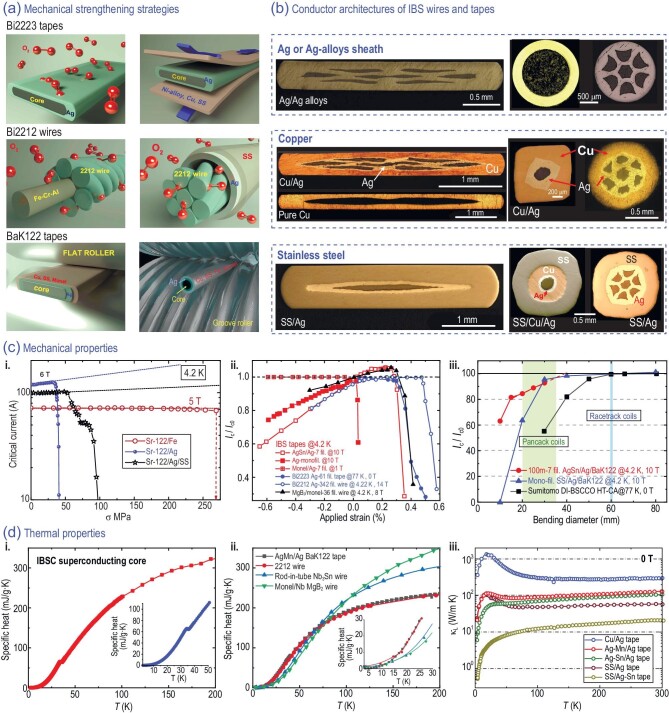
(a) Mechanical strengthening strategies of practical high-temperature superconductors. Top: lamination of Ni-alloy, Cu or SS on the Ag-sheathed Bi2223 tapes after annealing. Middle: the six-in-one cable (left) and the cable-in-conduit conductor structure (right) of Bi2212 wires. The red balls are oxygen molecules. Bottom: method of recombination and deformation used in BaK122 wires (right) and tapes (left). (b) Conductor architectures of IBS wires and tapes. The outer sheathes have been changed from Ag alloys [97] to copper [74,98,99] and SS [22,44]. Copyright 2020, IOP Publishing; Copyright 2022, Elsevier; Copyright 2023, Elsevier; Copyright 2023, IOP Publishing; Copyright 2021, IOP Publishing; Copyright 2016, IOP Publishing. (c) Mechanical properties of IBS tapes. i. Critical current as a function of tensile stress σ [100]. Copyright 2015, IOP Publishing. ii. Strain dependence of normalized critical current *I*_c_/*I*_c0_, where *I*_c0_ is the critical current before applying strain [92,101–104]. iii. *I*_c_ retention ratio of practical superconducting tapes after bending [105–107]. The green and blue areas mark the actual bending diameters of the fabricated pancake and racetrack IBS coils, respectively. The data of MgB_2_, Bi2223, and Bi2212 are also included in ii and iii. (d) Thermal properties of IBS tapes at 0 T. Temperature dependence of specific heat of (i) the superconducting core and (ii) the composite tapes [108]. The specific heat of Nb_3_Sn and MgB_2_ wires are included for comparison [109]. The insets enlarge the data at low temperatures. iii. Thermal conductivity of the IBS multifilamentary tapes as a function of temperature [97]. Copyright 2020, IOP Publishing.

The conductor architecture of practical superconducting wires and tapes is of vital importance to strengthen mechanical properties, improve thermal stability, decrease AC loss, and reduce material costs. For high-temperature superconducting PIT wires and tapes, expensive silver must be used as sheath materials. However, the hoop stress in high-field magnets described by $\sigma = BJR$ (*R* is the radius of solenoids) is usually several hundred MPa [85], much larger than the mechanical strength of Ag. Cuprate superconducting wires and tapes require an atmosphere with partial O_2_ pressure to allow oxygen diffusion into Ag or Ag-alloys, thereby modulating superconductivity. Consequently, the mechanical strength must be improved after annealing, e.g. the lamination technique of Bi2223 tapes used by Sumitomo Electric Industries, Ltd. [86], as shown in Fig. 8(a). For the Bi2212 wires, the 6-around-1 cable structure [87] and the cable-in-conduit conductor structure [88] were applied. The oxygen can pass through the interspace and contact the Ag sheath. So, the Bi2212 composite conductors can be annealed at the last step. On the contrary, the superconductivity of BaK122 is only controlled by K doping, no oxygen penetration is required. As a result, the outer part of the Ag sheath can be replaced by other metals or alloys, where the remaining Ag only acts as barrier layers to prevent reactions between BaK122 and the outer sheath (Cu, SS *et al.*). High-strength or high-conductive outer sheaths can be recombined and deformed along with the inner Ag wires and tapes before the final annealing. It not only varies the physical properties of the conductor but also makes a large difference to the microstructure of the filaments. Up to now, several outer sheath materials have been used, including SS [54,89], Cu [90], Fe [91], and Monel alloys [92]. Plus the number and arrangement of the filaments, there are multiple choices of conductor architectures for different application scenarios, as shown in Fig. 8(b).

The practical conductors applied in magnets are the ones that can be fabricated in long lengths at a low cost, carrying sufficient current. Kametani *et al.* estimated the cost per volume (liter) of BaK122 wires and tapes and compared it with state-of-the-art superconducting wires and tapes, as listed in Table 1 [93]. The selling price can be estimated by the final to raw material cost ratio, P. NbTi wires have a small P∼3 because of the easy and mature fabrication. The high price of YBCO coated conductors is caused by the expensive fabrication devices, low production yield and limited commercial market. This has recently been significantly alleviated by the development of scalable fabrication techniques and the increased demand from compacted fusion reactors [6]. Bi-based superconducting wires and tapes employ expensive Ag as sheath material and over-pressure (OP) heat treatment as the final annealing method, making the price the same level of REBCO (10^5^$/liter). We calculate the price of the BaK122 precursor powders (∼530$/kg), which are fabricated by the simple solid-state-reaction method, based on the price of the constituent elements, namely As (99.999%), Fe (99.9+%), Ba (99+%), and K (99%). It is only one-sixth of the price of 2212 precursors (∼3000$/kg) [94]. Moreover, the Ag sheath can be partially replaced by cheaper materials, leading to a further decrease in material cost. For instance, the Ag layer of the SS/Cu/Ag round wire in Fig. 8(b) is ∼70 μm thick and only takes ∼3% of the whole cross-sectional area. The P value can be further lowered because of the simpler PIT process than 2223 tapes and independence from high-pressure furnaces. As a result, scientists are optimistic about reducing the price of IBS wires and tapes to half the level of copper oxides [95] or even 4–5 times lower than that of Nb_3_Sn [96].

**Table 1.** Price ($/liter) and superconducting properties of candidate superconductors for high-field applications [93]. The IBS represented by BaK122 superconductors are highlighted in bold. The production-grade NbTi, Nb_3_Sn, Bi2212, REBCO wires and tapes are quoted here. Small high-field magnets fabricated at the National High Magnetic Field Laboratory (NHMFL) take one liter of conductors. The critical current density at 4.2 K and 20 T is present.

Material	P factor	Price per liter ($)	*T*_c_ (K)	*μ_0_H*_c2_ (4.2 K)	*J*_c_^intra^ (A/cm^2^)	*J*_c_^inter^ (A/cm^2^)	*J*_E_ (A/cm^2^)
Nb-Ti	3	∼2 × 10^3^	9.2	11 T	n/a	0	0
Nb_3_Sn	6–7	∼1.5 × 10^4^	18	26 T	n/a	∼4 × 10^4^	∼2.8 × 10^4^
Bi-2212	∼10	∼1 × 10^5^	85	∼100 T	n/a	∼6 × 10^5^	∼1.2 × 10^5^
REBCO	>>10	∼1 × 10^5^	92	>120 T	∼2 × 10^6^	∼2 × 10^6^	∼4.2 × 10^4^
**BaK122**	**∼10**	**< 4 × 10^3^**	**39**	**∼90 T**	**∼6 × 10^5^**	**∼10^4^–10^5^**	**∼(0.15–2)×10^4^**

Kováč *et al.* used a variable tensile load to measure the irreversible stress σ_irr_ of IBS tapes, as shown in Fig. 8(c)i. The σ_irr_ of the Ag sheathed tape is 35 MPa. It is enhanced to 50 MPa after being reinforced by double-sided SS tapes (40 μm). It is noteworthy that SS tapes are usually employed in magnet winding of iron-based tapes for reinforcement. The σ_irr_ of the iron sheathed tapes is over 270 MPa, implying better mechanical properties after applying a stronger sheath. Figure 8(c)ii shows the strain dependence of the normalized critical current measured by the U-spring. The Ag-sheathed IBS tapes show reversible compressive strain up to −0.65% [110]. The Monel/Ag sheathed composite tapes exhibit no *J*_c_ degradation even under a compressive strain of 0.6%, indicating enhanced strain tolerance of the strengthened structure [92]. The 100-m long 7 filament (fil.) AgSn/Ag/BaK122 tapes have a higher reversible tensile strain limit, ∼0.28%, than that of the mono-fil. tapes [104]. It is close to the 61-fil. Ag/Bi2223 tapes and 36 fil. MgB_2_/Monel wires [101–103]. Moreover, the *I*_c_ of IBS tapes is increased by 6% after applying a tensile strain. The bending diameter *R*_b_ of the 100-m long 7-fil. tapes co-wound with 0.1 mm SS tapes were measured via the bend-and-react method (similar to the coil fabrication process), as depicted in Fig. 8(c)iii. The ratio *I*_c_(bent tape)/*I*_c_(straight tape) decreases linearly from ∼92% at *R*_b_ = 30 mm to ∼80% at *R*_b_ = 15 mm. It promptly decreases to 63% at *R*_b_ = 10 mm, where obvious cracks can be found in the filaments [105]. The *I*_c_ retention of the SS/Ag mono-fil. tapes was measured after bending on a mandrel. There is no obvious drop until *R*_b_ < 30 mm [106]. Comparatively, the *I*_c_ of the Bi2223 tapes laminated by copper alloys after double-bending on a mandrel starts to decrease below 60 mm [107]. It seems that BaK122 tapes have better *I*_c_ retention than that of the Bi2223 tapes at the same bending diameter. However, it is noteworthy that different testing methods were applied.

The thermal-quench stability of long wires and tapes is vitally important for applications. Two crucial parameters, specific heat (*C*) and thermal conductivity (*κ*) of the wires and tapes are of great significance for magnet design. As shown in Fig. 8(d)i, the *C* of the bare superconducting core exhibits an obvious jump at T_c_ [108]. This jump is masked by the large *C* of the Ag sheath, as shown in Fig. 8(d)ii. The *C* of the IBS tapes is similar to that of the Bi2212 wire because of the similar Ag-based sheath materials. The thermal conductivity *κ* of the superconducting core is only ∼1–2 W/(m K). While the *κ* of the pure metal (Cu and Ag) is as large as ∼1000 W/(m K) [97,111]. The *κ* of metal sheaths is reduced by orders after impurity elements are doped. Consequently, the thermal properties of the wires and tapes are mainly determined by the sheath materials. For instance, the *κ* of the composite tapes can be changed from 1000 W/(m K) to 1 W/(m K) by modifying the conductor architectures (Fig. 8(d)iii). But precautions must also be taken to prevent elements’ interdiffusion across the sheath/sheath and the filament/sheath interfaces. Kováč *et al.* compared the AC loss of the mono- and 7-fil. BaK122 tapes via the calibration-free method [112]. It was found that the eddy current losses are significant in the highly conductive Ag-sheathed tapes. After the resistive AgSn outer sheath is applied, the eddy current loss is suppressed. This result also highlights the importance of choosing the appropriate sheath materials to diminish AC loss.

## APPLICATIONS OF IRON-BASED SUPERCONDUCTING LONG WIRES AND TAPES

**Figure 9.**
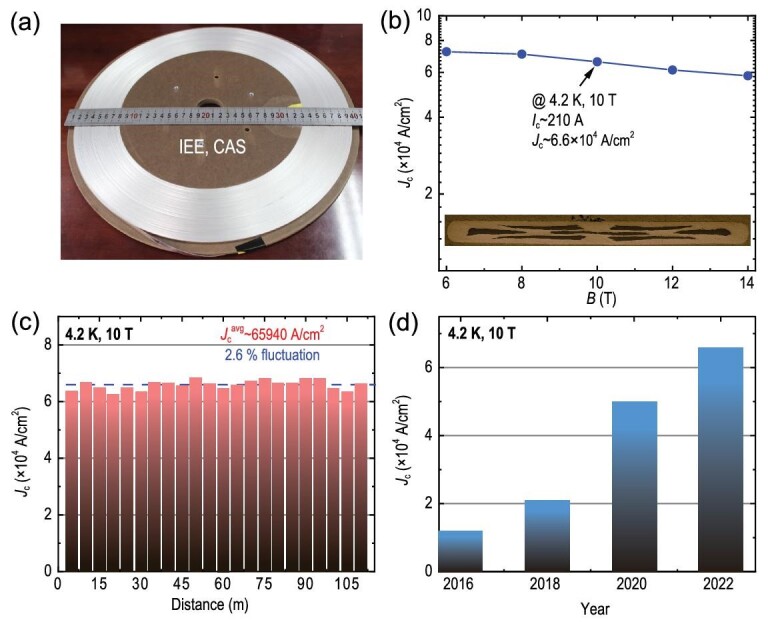
(a) 100-m long BaK122 tapes fabricated by the Institute of Electrical Engineering, Chinese Academy of Sciences (IEE, CAS). (b) Field dependence of *J*_c_. The inset shows the transverse cross-section of the tapes. (c) *J*_c_ homogeneity of the long tapes. (d) Development of *J*_c_(4.2 K, 10 T) of the 100-m long tapes since 2016.

The first 100-m long tapes were fabricated in 2016 by the scalable flat-rolling process [113]. After years of development and design, especially the advances in the quality and quantity of precursor powders, the performance of the 100-m long tapes has been progressively improved, as shown in Fig. 9(d). In 2022, the *J*_c_ of the 100-m long AgSn/Ag tapes had achieved 6.6 × 10^4^ A/cm^2^ (*I*_c_ = 210 A) at 4.2 K and 10 T. The long tapes exhibit a homogeneous *J*_c_ distribution with a small fluctuation of ∼2.6%.

**Figure 10.**
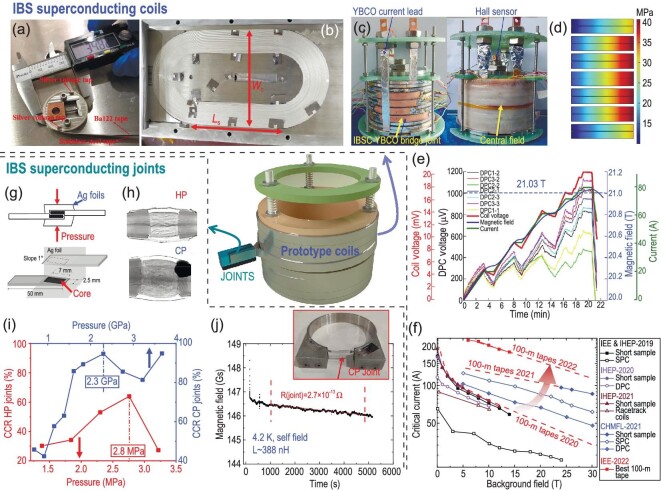
Iron-based superconducting coils. World's first (a) pancake coils [118] and (b) racetrack coils [119]. Copyright 2019, IOP Publishing; Copyright 2021, IOP Publishing. (c) The stack of seven DPC [120]. Copyright 2023, IOP Publishing. (d) Distribution of the Lorentz force on the stacked coils. (e) Charge test of the stacked coils. The colored thin lines correspond to the voltage of every DPC. (f) Field dependence of *I*_c_ of the 100-m long tapes and the superconducting coils [118,119,121,122]. The red dashed lines are the extrapolation of the *I*_c_-B of the 100-m long tapes. Iron-based superconducting joints. (g) Peeling-off joint (up) and angle-polishing joint (bottom) [115]. Copyright 2020, IOP Publishing; Copyright 2018, IOP Publishing. (h) XCT of the HP joint and the cold-pressed (CP) joint. Copyright 2019, IOP Publishing; Copyright 2022, IOP Publishing. (i) *I*_c_ retention rate of the HP and CP joints as a function of pressure [116,117]. The dash-dotted lines mark the optimal pressure. (j) Field-decay method for the resistance measurement of the joint [116]. Copyright 2022, IOP Publishing.

The persistent current operation of superconducting magnets in MRI and NMR systems requires superconducting joints with low resistance. The mono-fil. BaK122 superconducting joints have been fabricated by the peeling-off method or the angle-polishing method, as shown in Fig. 10(g) [114,115]. Then the exposed cores are joined face-to-face and wrapped by an Ag foil. The final joint was processed by HP or by sintering at AP after cold-pressing (CP). X-ray computed tomography indicates a better connection in the CP joints (Fig. 10(h)) [116]. The critical current ratio (CCR = *I*_c_^joint^/*I*_c_^tape^) shown in Fig. 10(i) ascends quickly to the maximum at optimal pressure. The best CCR achieved in the CP joints is 94.6%, much higher than that of the HP joints [116,117]. Zhu *et al.* utilized the field-decay method to measure the CP joint in a BaK122 closed-loop coil. The persistent current is 132.7 A after the test. A low joint resistance of 2.7 × 10^−13^ Ω is obtained at 4.2 K and self-field, indicating the good application potential of BaK122 joints in high-field magnets.

Several kinds of IBS coils have already been fabricated from the long wires and tapes, as summarized in Table 2. All the coils were made by the wind-and-react method. The final sintering process was generally performed in an Ar atmosphere at AP except for the solenoid coils sintered by HIP. The tapes of the pancake and racetrack coils were wound in parallel with the SS tapes because most insulation materials cannot withstand sintering temperatures above 800°C [119]. The inside diameter of the single (SPC) and double pancake coils (DPC) is generally ∼30–35 mm, corresponding to >90% *I*_c_ retention of the unbent samples (Fig. 8(c)iii). Li *et al.* fabricated DPC with an inside diameter of ∼20 mm, which corresponds to 84.4% *I*_c_ retention, to increase the turns to 19 [123]. Multiple tests in liquid helium cause no degradation of performance. Solenoid coils were made from the Cu/Ag wires by the HIP method at 700°C [69,98]. Fiberglass sleeves were used for insulation. The generated field at 0 T is 0.26 T, which is limited by the inhomogeneous *J*_c_ distribution. Worthy of note is that seven IBS DPC made from the ∼260 m long IBS tapes were recently stacked and connected with YBCO bridge joints to fabricate an insert coil [120]. It generates a 1.03 T magnetic field under a background field of 20 T at 4.2 K. The calculated maximum radial Lorenz force is ∼40.6 MPa (Fig. 10(d)), exceeding the yield strength of the Ag-sheathed tapes [100]. It may result in a degraded *I*_c_ of one DPC. A higher field would be expected after applying high-strength IBS tapes. Figure 10(f) summarizes the *I*_c_ – *B* curves of the short samples and the coils. The red lines corresponding to the *I*_c_ of the 100-m long tapes demarcate the figure into several regions. (See online supplementary material for a color version of this figure.) The performance of the coils was improved by optimizing the fabrication technique but also limited by the *I*_c_ and homogeneity of the long tapes. For now, the highest reported current of the pancake coils is 139 A at 10 T and 67 A at 30 T, which are ∼77% and 76% of the *I*_c_ of the short samples, respectively [123]. While the *I*_c_ of the racetrack coils achieves 87% of the *I*_c_ of the short samples [119]. The future improvement of *I*_c_ in long tapes would undoubtedly contribute to higher performance coils. The design, fabrication and test of prototypes of IBS superconducting coils represent one big step into practical applications and demonstrate the great potential of IBS long wires and tapes at high fields.

**Table 2.**
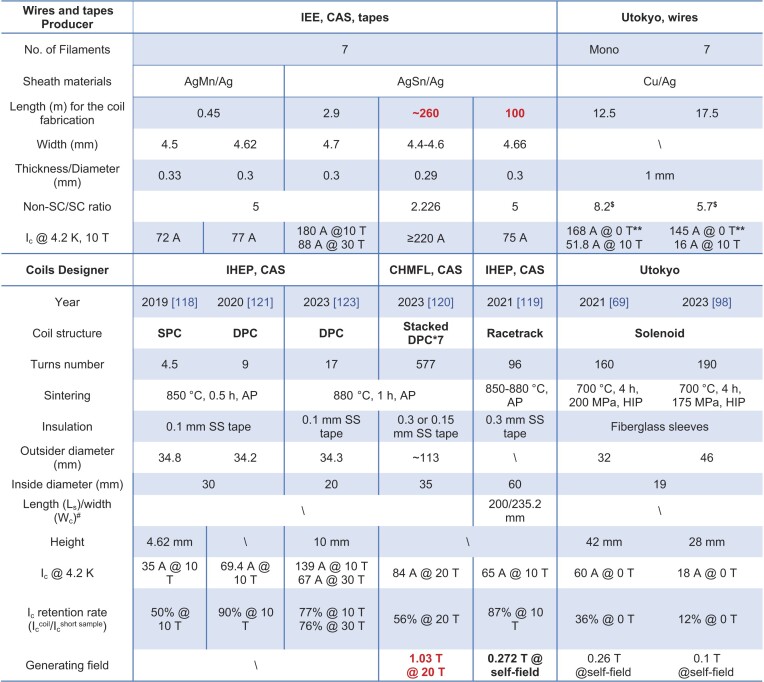
IBS wires, tapes and coils. The coils are made from the wires and tapes listed in the same column. The designers and manufacturers are the IEE, CAS, the Institute of High Energy Physics (IHEP), the Chinese High Magnetic Field Laboratory (CHMFL), and the University of Tokyo (Utokyo). (**^#^**L_s_ and W_c_ are marked in Fig. 10 (b). **^$^**The non-SC/SC ratio of the wire is calculated from the transverse cross-section in refs. [69,98]. **The *I*_c_ is measured on the short segments from the coils.)

## CONCLUSIONS AND PROSPECTS

Future high-field applications require multiple advanced characteristics of practical superconductors, among which the current carrying ability and the fabrication cost are two top priorities. Although the high *J*_c_–10^6^∼10^7^ A/cm^2^ has already been achieved in copper-oxide superconducting wires and tapes, the price is greatly limited by fabrications and raw materials. On the contrary, low cost is one of the appealing attributes of IBS wires and tapes. The easy PIT process, the earth-abundant element materials, and the independence from the Ag sheath allow IBS wires and tapes to be cheaper than Nb_3_Sn. After one decade of research and design, the *J*_c_ of BaK122 tapes has made significant progress and exhibits nearly isotropic field dependence. The best *J*_c_(4.2 K, 10 T) of the short and long BaK122 PIT tapes have achieved 2.6 × 10^5^ A/cm^2^ and 6.6 × 10^4^ A/cm^2^, respectively. There is still significant upside potential for the current carrying capacity. Besides, the mechanical properties, AC-loss characteristics, and thermal stability can be tailored by modifying the conductor architectures, such as sheath materials and filament arrangements, to meet the stringent requirements of high-field applications. For now, the 100-m long tapes have been fabricated and applied in the prototypes of IBS insert coils. They generate over 1 T magnetic field under a background field of 20 T, indicating competitive potential in high-field applications. The 1000-m long iron-based superconducting wire project has been proposed and commenced by the IEE, CAS. This is a big progress for the industrialization and pervasive applications of IBS. But still, there are many theoretical and technical open questions for the further development of high-performance IBS wires and tapes.

1. **Massive production of high-quality precursor powders**. The key challenge is to figure out the formation mechanism of impurities, especially FeAs and oxides, and how to avoid them.
2. **Texture and current transport model**. Currently, only partial *c*-axis texture has been realized in the tape form. Further enhancing the *c*-axis texture or even introducing local in-plane texture is expected to significantly improve *J*_c_. Based on the grain morphology and alignment, an intrinsic current transport model could be developed to inspire the next strategy to enhance *J*_c_.
3. **Flux pinning**. After the grain coupling is improved, the intra-grain and inter-grain flux pinning becomes more important. Irradiation seems to be an easy way to introduce strong flux pinning centers. But plenty of technical issues remain to be solved. For instance, thick metal sheaths (100–300 μm) limit the option to protons or neutrons, but meanwhile, radioactivity cannot be avoided in a short time.
4. **Large-scale production of high-strength composite tapes**. Homogeneous deformation of high-strength composite wires and tapes is difficult because of the asynchronous transformation of the different sheaths and the superconducting cores. Finite element simulation can play an important part in saving time and cost through trial and error. Moreover, strain and stress data of the composite wires and tapes are highly desired for magnet design.
5. **Joints, coils, and cables**. Multifilament wires and tapes are preponderant in future applications. However, fabricating high-performance joints between them is a difficult task. The design and manufacture of IBS coils must be optimized, especially when high-strength composite wires and tapes are applied. IBS cables, e.g. the transposed cables and the Rutherford cables, should also be studied due to their high current carrying capacity, low dynamic loss, and high mechanical stability.

After the above-mentioned challenges are fully addressed, we believe that the high-performance, low-cost iron-based superconducting wires and tapes will be widely used in fusion reactors, next-generation accelerators, and advanced MRI and NMR systems.
